# Periodontal Disease and Bacterial Vaginosis Increase the Risk for Adverse Pregnancy Outcome

**DOI:** 10.1155/2005/642939

**Published:** 2005-12-22

**Authors:** Juha Oittinen, Tapio Kurki, Minnamaija Kekki, Minna Kuusisto, Pirkko Pussinen, Tiina Vilkuna-Rautiainen, Anja Nieminen, Sirkka Asikainen, Jorma Paavonen

**Affiliations:** ^1^ Institute of Dentistry, Oral and Maxillofacial Diseases, University of Helsinki, Biomedicum Helsinki, Helsinki, Finland; ^2^ University of Helsinki, Department of Obstetrics and Gynecology, Haartmaninkatu 2, Helsinki 00290, Finland; ^3^ NDivision of Oral Microbiology, Faculty of Medicine and Odontology, Umeå University, Umeå, Sweden

## Abstract

*Objective*. To determine whether periodontal disease or bacterial vaginosis (BV) diagnosed before pregnancy increase the risk for adverse pregnancy outcome.

*Methods*. We enrolled a total of 252 women who had discontinued contraception in order to become pregnant. The first 130 pregnant women were included in the analyses.

*Results*. Multivariate analysis showed a strong association between periodontal disease and adverse pregnancy outcome (OR 5.5, 95% confidence interval 1.4–21.2; p = 0.014), and a borderline association between BV and adverse pregnancy outcome (OR 3.2, 95% confidence interval 0.9–10.7; p = 0.061).

*Conclusions*. TOur study suggests that pre-pregnancy counseling should include both oral and vaginal examinations to rule out periodontal disease and BV. This may ultimately have an impact on antenatal healthcare, and decrease the risk for adverse pregnancy outcome.


*Infectious Diseases in Obstetrics and Gynecology*, December 2005; 13(4): 213–216

Taylor & Francis

Taylor & Francis Group

**CLINICAL STUDY**

### Periodontal disease and bacterial vaginosis increase the risk for adverse pregnancy outcome

JUHA OITTINEN[1], TAPIO KURKI[2], MINNAMAIJA KEKKI[2], MINNA KUUSISTO[2], PIRKKO PUSSINEN[1], TIINA VILKUNA-RAUTIAINEN[1], ANJA NIEMINEN[1], SIRKKA ASIKAINEN[3], & JORMA PAAVONEN[2]

[1]*Institute of Dentistry, Oral and Maxillofacial Diseases, University of Helsinki, Biomedicum Helsinki, Helsinki, Finland,* [2]*Department of Obstetrics and Gynecology, University of Helsinki, Finland, and* [3]*Division of Oral Microbiology, Faculty of Medicine and Odontology, Umeå University, Umeå, Sweden*

*(Received 19 January 2005; accepted 27 April 2005)*

**Abstract**

*Objectives.* To determine whether periodontal disease or bacterial vaginosis (BV) diagnosed before pregnancy increase the risk for adverse pregnancy outcome.

*Methods.* We enrolled a total of 252 women who had discontinued contraception in order to become pregnant. The first 130 pregnant women were included in the analyses.

*Results.* Multivariate analysis showed a strong association between periodontal disease and adverse pregnancy outcome (OR 5.5, 95% confidence interval 1.4–21.2; $p = 0.014$), and a borderline association between BV and adverse pregnancy outcome (OR 3.2, 95% confidence interval 0.9–10.7; $p = 0.061$).

*Conclusion.* Our study suggests that pre-pregnancy counseling should include both oral and vaginal examinations to rule out periodontal disease and BV. This may ultimately have an impact on antenatal healthcare, and decrease the risk for adverse pregnancy outcome.

**Keywords:** *Periodontal disease, bacterial vaginosis, adverse pregnancy outcome*

### Introduction

Known risk factors for preterm delivery include previous preterm birth, multiple births, low socio-economic status, low education, low maternal weight, smoking, alcohol consumption [1,2], and specific genitourinary tract infections [3]. However, attempts to reduce the rate of preterm delivery based on risk scoring systems have largely failed [4]. Often the cause of preterm delivery remains unknown.

Maternal infections, such as periodontitis and bacterial vaginosis (BV) have been linked to an increased risk for adverse pregnancy outcome. BV has been consistently associated both with miscarriage and preterm delivery [5,6]. However, intervention trials on treatment of BV during pregnancy have largely failed to reduce the rates of preterm birth [7,8] or only marginally reduced the rates [9,10]. Recent studies have demonstrated that maternal periodontal disease diagnosed during pregnancy also increases the risk for low birth weight and prematurity [11–16] even though contradictory results have also been reported [17]. Furthermore, periodontal therapy may reduce the risk for adverse pregnancy outcome [18,19]. These observations are not unexpected since both BV and periodontal disease are associated with high bacterial burden and increase of pro-inflammatory cytokines and other mediators of inflammation [19,20]. We conducted a prospective study among women planning pregnancy to clarify whether periodontal disease or BV diagnosed before pregnancy increase the risk for adverse pregnancy outcome.

Correspondence: Jorma Paavonen, MD, University of Helsinki, Department of Obstetrics and Gynecology, Haartmaninkatu 2, 00290 Helsinki, Finland. Tel: +358 9 4 717 2807. Fax: +358 9 4 717 4902. E-mail: jorma.paavonen@hus.fi

ISSN 1064-7449 print/ISSN 1098-0997 online © 2005 Taylor & Francis
DOI: 10.1080/10647440500240730

### Material and methods

A total of 256 healthy Finnish women were enrolled by newspaper advertisements, between May 2001 and July 2004. All women were Caucasian, not pregnant, menstruating regularly, had discontinued contraception and wanted to get pregnant. Exclusion criteria included history of preterm delivery and use of antibiotics within the preceding 2 weeks. The study protocol was approved by the Ethics Committee of the Department of Obstetrics and Gynecology, University of Helsinki. Each subject signed an informed consent. The first 130 women who became pregnant were included in this report.

The medical history was systematically collected. Oral and gynecological examinations were performed before pregnancy. The investigator (J.O.) performing oral examinations was blinded to the results obtained by gynecological examinations. Oral examination included recording the presence of caries lesions extending to dentin, and the presence of periodontal disease (periodontal pocket depth, gingival bleeding on probing, probing attachment loss at six sites per tooth). Periodontal disease was diagnosed when at least one approximal periodontal pocket was $\geqslant 4$ mm and attachment loss $\geqslant 1$ mm.

Gynecological speculum examination was performed before pregnancy and again during the first trimester (between 6 and 8 weeks of gestation), and twice during the third trimester (between 28 and 32 weeks of gestation). A vaginal swab was taken and Gram-stained for the diagnosis of BV [7]. The diagnosis of BV was based on validated Nugent's criteria [21]. Miscarriage was defined as pregnancy loss before 22 weeks of gestation. Preterm delivery was defined as birth before 37 weeks of gestation.

The statistical significance of differences in frequency distributions between groups was tested by the Chi-square test and the Fisher's exact test; means of continuous variables by the Mann–Whitney U-test. Logarithmic transformation was used for skewed data. Univariate analyses and multivariate logistic regression analyses were performed. A $p$-value less than 0.05 was considered significant.

### Results

The 130 women who became pregnant did not differ from the total group of 256 women regarding demographic variables or findings on oral and gynecological examinations. A total of 26 (20%) women had an adverse pregnancy outcome, including 17 women with miscarriage and nine women with preterm birth. The mean gestational age in cases of preterm delivery was 34 weeks (range 30–36 weeks). Women with or without adverse pregnancy outcome did not differ by age, socioeconomic status, smoking, or general health problems (data not shown). Periodontal disease (defined as at least one inflamed periodontal pocket $\geqslant 4$ mm with periodontal attachment loss $\geqslant 1$ mm) was found in 15 (12%) women. BV was found in 21 (16%) women. By univariate analyses, periodontal disease ($p = 0.012$) and BV ($p = 0.014$) were the only variables significantly associated with adverse pregnancy outcome (data not shown). Multivariate analyses showed that periodontal disease was significantly associated with adverse pregnancy outcome (OR 5.5, 95% CI 1.4–21.2, $p = 0.014$) (Table I). BV showed a borderline association with adverse pregnancy outcome (OR 3.2, 95% CI 0.9–10.7, $p = 0.061$). Of the five women with both periodontal disease and BV, three developed adverse pregnancy outcome (OR 13.1, 95% CI 1.9–135,1, $p = 0.031$).

### Discussion

We showed that periodontal disease diagnosed prior to pregnancy was an independent predictor of adverse pregnancy outcome. Thus, the results of our prospective study emphasize the role of chronic periodontal disease as a new risk factor for adverse pregnancy outcome. Previous studies have also suggested an association between periodontal disease and preterm delivery [11–16], as well as demonstrating a reduced rate of preterm birth after periodontal therapy [11,12]. Many earlier studies have shown a strong link between BV and miscarriage or preterm birth [18,19].

The mechanisms by which periodontal disease may cause pregnancy loss or preterm birth remain speculative. Increased bacterial burden or qualitative change of the microbial flora in inflamed periodontal pockets may lead to hematogenous spread of microorganisms, their components, or proinflammatory mediators [19]. In some women this may then cause decidual infection or inflammation leading to adverse pregnancy outcome.

BV is known to cause subclinical endometritis or deciduitis with ascension of virulent BV-associated pathogens to the upper genital tract [3]. In this study, BV was also associated with adverse pregnancy outcome by univariate analyses. However, the most striking finding was that by multivariate analyses this association was only of borderline significance.

The strength of our study was that it was prospective. None of the previous case-control or cohort studies of the risk factors or risk markers for preterm birth have simultaneously analysed the role of periodontal disease and BV.

Our preliminary data suggest that BV may further increase the risk for adverse pregnancy outcome

Table I. Multivariate analysis of risk factors for adverse pregnancy outcome in women planning pregnancy.

| Variable | *N* | (%) | OR (95% CI) | *p*-Value |
|---|---|---|---|---|
| Periodontal breakdown* | | | | |
| No | 115 | (88) | 1.0 (reference group) | |
| Yes | 15 | (12) | 5.5 (1.4–21.2) | 0.014 |
| Bacterial vaginosis | | | | |
| No | 109 | (84) | 1.0 (reference group) | |
| Yes | 21 | (16) | 3.2 (0.9–10.7) | 0.061 |
| Social class[†] | | | | |
| I–II | 36 | (28) | 1.0 (reference group) | |
| III–IV | 94 | (72) | 0.3 (0.1–1.2) | 0.086 |
| Parous | | | | |
| Yes | 75 | (58) | 1.0 (reference group) | |
| No | 55 | (42) | 0.4 (0.1–1.2) | 0.087 |
| History of induced abortion | | | | |
| No | 117 | (90) | 1.0 (reference group) | |
| Yes | 13 | (10) | 3.0 (0.6–15.8) | 0.202 |
| Continuous medication | | | | |
| No | 106 | (82) | 1.0 (reference group) | |
| Yes | 24 | (18) | 1.7 (0.4–6.5) | 0.446 |
| Antimicrobial treatment[‡] | | | | |
| No | 78 | (60) | 1.0 (reference group) | |
| Yes | 52 | (40) | 0.7 (0.2–2.1) | 0.494 |
| Smoker | | | | |
| No | 113 | (87) | 1.0 (reference group) | |
| Yes | 17 | (13) | 0.6 (0.1–3.0) | 0.549 |
| General health problems | | | | |
| No | 122 | (94) | 1.0 (reference group) | |
| Yes | 8 | (6) | 1.6 (0.2–11.7) | 0.638 |
| Allergy | | | | |
| No | 108 | (83) | 1.0 (reference group) | |
| Yes | 22 | (17) | 1.3 (0.4–4.7) | 0.711 |
| Treatment for infertility | | | | |
| No | 124 | (95) | 1.0 (reference group) | |
| Yes | 6 | (5) | 1.3 (0.1–14.1) | 0.823 |
| Age (increase per year) | 130 | (100.0) | 1.0 (0.9–1.1) | 0.825 |
| Any gynecological infection | | | | |
| No | 76 | (59) | 1.0 (reference group) | |
| Yes | 54 | (41) | 1.1 (0.4–3.1) | 0.883 |
| History of miscarriage | | | | |
| No | 95 | (73) | 1.0 (reference group) | |
| Yes | 35 | (27) | 1.1 (0.3–3.6) | 0.928 |
| Caries disease | | | | |
| No | 101 | (78) | 1.0 (reference group) | |
| Yes | 29 | (22) | 1.0 (0.3–3.4) | 0.997 |

*Periodontal attachment loss in inflamed periodontal pockets. [†]Social class divided into two categories according to the woman's profession (Low = non-skilled laborer or skilled laborer; High = non-academic white-collar or academic profession). [‡]No microbial treatment within preceding 2 weeks.

among women with periodontal disease. Thus, periodontal disease and BV may have additive effects. However, the total number of true endpoints in our study is still relatively low.

One of the key questions clearly is to sort out the link between periodontal disease and BV. Preliminary data from our study suggest that BV and periodontal disease are interrelated [22]. Women with BV may be more likely to practice receptive oral sex which may in turn predispose them to periodontal disease. Alternatively, male partners with periodontal disease may predispose women to BV or periodontal disease, or both [23]. Larger studies are needed to explore this.

In Finland, routine dental examination has been recommended to all pregnant women, although not all women seek dental care. Our results, if confirmed, would suggest that pre-pregnancy counseling should include both oral and vaginal examinations to rule out periodontal disease and BV. This may ultimately have a major impact on antenatal healthcare, and may be a new intervention to decrease the risk for unexplained or idiopathic adverse pregnancy outcome.

### Acknowledgments

This study was supported by research grants from the Helsinki University Research Funds (TYH 2239, TYH 4261, T1030A0029, T1030A0050), Umeå University Research Funds, Paulon Säätiö, and The Finnish Dental Society Apollonia. The sponsors of the study had no role in study design, data collection, data analysis, data interpretation, or in the writing of this report.

### References

1. Kramer MS, Seguin L, Lydon J, Goulet L. Socio-economic disparities in pregnancy outcome: why do the poor fare so poorly? Paediat Perinat Epidemiol 2000;14:194–210.
2. Slattery MM, Morrison JJ. Preterm delivery. Lancet 2002;360:1489–1497.
3. Goldenberg RL, Hauth JC, Andrews WW. Intrauterine infection and preterm delivery. N Engl J Med 2000;342: 1500–1507.
4. Mercer B, Goldenberg R, Das A, Moawad A, Iams J, Meis P, Copper R, Jol F, Thom E, McNellis D. The preterm prediction study: a clinical risk assessment system. Am J Obstet Gynecol 1996;174:1885–1895.
5. Oakeshott P, Hay P, Hay S, Steinke F, Rink E, Kerry S. Association between bacterial vaginosis or chlamydial infection and miscarriage before 16 weeks' gestation: prospective community based cohort study. BMJ 2002;325:1334–1338.
6. Leitich H, Bodner-Adler B, Brunbauer M, Kaider A, Egarter C, Husslein P. Bacterial vaginosis as a risk factor for preterm delivery: A meta-analysis. Am J Obstet Gynecol 2003;189: 139–147.
7. Kekki M, Kurki T, Pelkonen J, Kurkinen-Räty M, Cacciatore B, Paavonen J. Vaginal clindamycin in preventing preterm birth and peripartal infections in asymptomatic women with bacterial vaginosis: a randomized, controlled trial. Obstet Gynecol 2001;97:643–648.
8. Carey JC, Klebanoff MA, Hauth JC, Hillier SL, Thom EA, Ernest JM, Heine RP, Nugent RP, Fischer ML, Leveno KJ, et al. Metronidazole to prevent preterm delivery in pregnant women with asymptomatic bacterial vaginosis. N Engl J Med 2000;342:534–540.
9. Ugwumadu A, Manyonda I, Reid F, Hay P. Effect of early oral clindamycin on late miscarriage and preterm delivery in asymptomatic women with abnormal vaginal flora and bacterial vaginosis: a randomized controlled trial. Lancet 2003;361:983–988.
10. Kiss H, Petricevic L, Husslein P. Prospective randomized controlled trial of an infection screening programme to reduce the rate of preterm delivery. BMJ 2004;329:371–374.
11. Offenbacher S, Katz V, Fertig G, Collins J, Boyd D, Maynor G, McKaig R, Beck J. Periodontal infection as a possible risk factor for preterm low birth weight. J Periodontol 1996;7: 1103–1113.
12. Offenbacher S, Javed HL, O'Reilly PG, Wells SR, Salvi GE, Lawrence HP, Socransky SS, Beck JD. Potential pathogenic mechanism of periodontitis-associated pregnancy complications. Ann Periodontol 1998;3:233–250.
13. Offenbacher S, Lieff S, Boggess K, Murtha AP, Madianos PN, Champagne CM, McKaig RG, Jared HL, Mauriello SM, Hauten RL, et al. Maternal periodontitis and prematurity. Part I: Obstetric outcome of prematurity and growth restriction. Ann Periodontol 2001;6:164–174.
14. Madianos PN, Lieff S, Murtha KA, Boggess KA, Auten RL, Beck JD, Offenbacher S. Maternal periodontitis and prematurity. Part II: Maternal infection and fetal exposure. Ann Periodontol 2001;6:175–182.
15. Jeffcoat MK, Geurs RC, Reddy MS, Goldenburg RL, Hauth JC. Current evidence regarding periodontal disease as a risk factor in preterm birth. Ann Periodontol 2001;6:183–188.
16. Collins J, Windley III H, Arnold R, Offenbacher S. Effects of a *Porphyromonas gingivalis* infection on inflammatory mediator response and pregnancy outcome in hamsters. Infect Immun 1994;62:4356–4361.
17. Davenport ES, Williams CE, Sterne JA, Murad JS, Sivapathasundram V, Curtis MA. Maternal periodontal disease and preterm low birthweight: Case-control study. J Dent Res 2002;81:313–318.
18. Jeffcoat MK, Hauth JC, Geurs NC, Reddy MS, Cliver SP, Hodgkins PM, Goldenburg RL. Periodontal disease and preterm birth: Results of a pilot intervention study. J Periodontol 2003; 74:1214–1218.
19. Lopez N, Smith P, Gutierrez J. Periodontal therapy may reduce the risk of preterm low birth weight in women with periodontal disease: a randomized controlled trial. J Periodontol 2002;73:911–924.
20. Andrews WW. Cervicovaginal cytokines, vaginal infection, and preterm birth. Am J Obstet Gynecol 2004;190:1179.
21. Nugent RP, Krohn MA, Hillier SL. Reliability of diagnosing bacterial vaginosis is improved by a standardized method of Gram stain interpretation. J Clin Microbiol 1991;29:297–301.
22. Oittinen J, Kurki T, Pussinen PJ, Vilkuna-Rautiainen T, Kekki M, Kuusisto M, Nieminen A, Paavonen J, Asikainen S. Periodontitis before pregnancy increases the risk for adverse pregnancy outcome. IADR 81st General Session in Goteborg, Sweden, 2003. J Dent Res 2003;82(Special Issue B):0125.
23. Saarela M, von Troil-Linden B, Torkko H, Stucki AM, Alaluusua S, Jousimies-Somer H, Asikainen S. Transmission of oral bacterial species between spouses. Oral Microbiol Immunol 1993;8:349–354.

